# Complete and Draft Genome Sequences of 48 Staphylococcus aureus Isolates Obtained from Atopic Dermatitis Patients and Healthy Controls

**DOI:** 10.1128/mra.00072-22

**Published:** 2022-03-08

**Authors:** Zhongjie Wang, Claudia Hülpüsch, Vera Schwierzeck, Sulaiman Ali Alharbi, Matthias Reiger, Claudia Traidl-Hoffmann, Michael Schloter, Bärbel U. Foesel

**Affiliations:** a Research Unit for Comparative Microbiome Analysis, Helmholtz Center Munich, German Research Center for Environmental Health, Neuherberg, Germany; b Department of Environmental Medicine, Faculty of Medicine, University of Augsburg, Augsburg, Germany; c Department of Botany & Microbiology, College of Science, King Saud University, Riyadh, Saudi Arabia; d Technical University of Munich, Central Institute for Food and Health (ZIEL), Freising, Germany; University of Rochester School of Medicine and Dentistry

## Abstract

Staphylococcus aureus is a widely distributed, opportunistic pathogen and has been linked to the human skin disease atopic dermatitis (AD). Here, we present 44 complete and 4 draft genome sequences of S. aureus strains isolated from the nose and skin of AD patients and healthy controls from a German study cohort.

## ANNOUNCEMENT

Staphylococcus aureus is an integral part of the human microbiome (1), colonizing the nasopharynx and skin. S. aureus can also act as an opportunistic pathogen (2), playing a role in atopic dermatitis (AD) (3, 4).

Here, we report genome sequences of 48 S. aureus strains (44 genomes with complete and 4 with incomplete chromosomes) isolated from the skin and nose of 10 subjects from a study cohort established at the Klinikum Augsburg in Germany, approved by the ethics committee of the Technical University of Munich (112/16 S and 187/17 S).

For isolation of S. aureus, skin and nose swabs (Transwabs; Medical Wire, Wiltshire, England) were sampled from June 2018 to March 2019; potential S. aureus isolates were retrieved by initial streaking and subsequent purification on mannitol-salt agar plates (Carl Roth, Karlsruhe, Germany). Identity was confirmed by matrix-assisted laser desorption ionization–time of flight (MALDI-TOF) after growth on Columbia blood agar plates with 5% sheep blood (Oxoid Ltd., Basingstoke, UK). The Genomic-tip kit 20/G (Qiagen, Hilden, Germany) was used for genomic DNA extraction from approximately 4.5 × 109 cells (equivalent to an optical density at 600 nm [OD_600_] of 0.5) freshly grown in LB broth (Carl Roth) at 37°C for approximately 4 to 5 h. Cells were harvested by centrifugation at 3,270 × *g* for 10 min. For extraction, lysozyme was replaced by lysostaphin (20 μL of a 10-mg/mL stock solution).

Genome sequencing was carried out on a PacBio Sequel platform (Pacific Biosciences, Menlo Park, CA) using the Sequel binding kit v3.0, sequencing plate v3.0 (4 reaction), and SMRT cell 1M v2 (PacBio). Library preparation was performed by applying the SMRTbell Express template prep kit v2.0 and the barcoded adapter kit v8A (PacBio) according to the protocol provided by the manufacturer. Genomic DNA was sheared to ∼10 kb using g-TUBEs (Covaris, Inc., Woburn, MA) and processed without additional size selection. SMRTBell libraries (4 pools, each including 12 strains) were loaded according to the diffusion loading protocol (PacBio) at a concentration of 5 pm. Movie time was 10 h per SMRT cell after immobilization for 2 h and preextension for 2 h.

After data demultiplexing, genome assembly was performed with the HGAP v4 pipeline as embedded in SMRTLink v6.0.0.47841 (PacBio). For the remaining incomplete assemblies, the genome assembly and high-quality circular consensus sequence (CCS) reads generated by SMRTLink were used for circularization using Circlator v1.5.5 with the parameter --merge_min_length_merge 1000 (5).

All complete chromosomal scaffolds were rotated to *dnaA* as the start position. If no *dnaA* gene was found for the incomplete assemblies, then Prodigal v2.6.3 (6) was used to identify the gene nearest the center of the contig. Genome qualities were assessed using CheckM v1.1.3 (7); assemblies were annotated by deploying the NCBI Prokaryotic Genome Annotation Pipeline (PGAP) (8). Default parameters were used for all software unless otherwise specified.

Overall genome sizes ranged from 2.7 to 3.1 Mb, with individual genomes displaying 1 chromosomal and 0 to 6 additional plasmid scaffolds. The G+C content ranged from 32.7 to 33.0%. The genomes contained 2,500 to 2,818 predicted coding sequences. The number of tRNAs detected ranged from 56 to 65. Details of the assembly statistics and source information are listed in Table 1.

**TABLE 1:** Genome assembly statistics and source information of the 48 Staphylococcus aureus isolates

Strain	GenBank assembly accession no.	BioSample accession no.	Genome size (bp)	No. of scaffolds	*N*_50_ (bp)	GC (%)	No. of coding genes	No. of tRNAs	Chromosome	Contamination (%)	No. of realigned subreads	Avg subread length (bp)	Genome coverage (×-fold)	Skin location	Health status^*a*^
194	CP077932	SAMN19550019	2,754,399	1	2,754,399	33	2,562	59	Complete	0.13	120,834	4,943	326	Skin	AE
195	CP077931	SAMN19550020	2,754,414	1	2,754,414	33	2,556	59	Complete	0.13	120,639	4,884	667		
196	CP077928 CP077929 CP077930	SAMN19550021	2,828,046	3	2,754,404	33	2,642	59	Complete	0.97	98,907	5,164	467		
197	CP077926 CP077927	SAMN19550022	2,788,328	2	2,754,402	33	2,573	59	Complete	0.13	135,207	4,965	289	Nose	
198	CP077925	SAMN19550023	2,754,401	1	2,754,401	33	2,564	59	Complete	0.13	138,422	4,638	442		
199	CP077939 CP077940	SAMN19550024	2,793,031	2	2,764,397	33	2,585	65	Draft	1.54	179,644	5,099	271		
200	CP077924	SAMN19550025	2,719,166	1	2,719,166	33	2,500	59	Complete	0.08	169,836	4,607	526	Nose	HE
201	CP077923	SAMN19550026	2,719,235	1	2,719,235	33	2,502	59	Complete	0.08	206,880	5,115	432		
202	CP077922	SAMN19550027	2,719,233	1	2,719,233	33	2,505	59	Complete	0.08	196,190	5,243	525		
276	CP077921	SAMN19550028	2,730,804	1	2,730,804	32.9	2,537	59	Complete	0.08	281,434	5,163	483	Skin	AE
277	CP077920	SAMN19550029	2,749,120	1	2,749,120	32.9	2,546	59	Complete	0.11	181,163	4,818	259		
278	CP077918 CP077919	SAMN19550030	2,785,715	2	2,732,781	32.8	2,585	59	Complete	1.77	178,032	5,263	203		
280	CP077917	SAMN19550031	2,748,827	1	2,748,827	32.9	2,548	56	Complete	0.11	95,473	5,487	251	Nose	
281	CP077916	SAMN19550032	274,8821	1	2,748,821	32.9	2,545	56	Complete	0.11	200,607	5,157	192		
282	CP077915	SAMN19550033	2,732,780	1	2,732,780	32.9	2,539	59	Complete	0.08	152,840	4,691	395		
317	CP077913 CP077914	SAMN19550034	2,955,403	2	2,911,834	32.8	2,767	56	Draft	0.25	232,973	5,074	219	Nose	HE
318	CP077911 CP077912	SAMN19550035	2,952,667	2	2,919,985	32.8	2,740	56	Complete	2.5	209,420	5,129	329		
319	CP077936 CP077937 CP077938	SAMN19550036	2,952,363	3	2,890,321	32.8	2,754	56	Complete	0.62	259,002	5,765	253		
322	CP077908 CP077909 CP077910	SAMN19550037	2,831,656	3	2,764,234	32.8	2,646	59	Complete	0.13	316,777	4,789	316	Nose	HE
323	CP077906 CP077907	SAMN19550038	2,873,158	2	2,828,011	32.9	2,644	59	Draft	3.25	497,238	5,126	216		
324	CP077899 CP077900 CP077901 CP077902 CP077903 CP077904 CP077905	SAMN19550039	3,123,465	7	2,816,989	32.8	2,818	59	Draft	4.15	342,044	5,027	194		
327	CP077898	SAMN19550040	2,764,236	1	2,764,236	33	2,594	59	Complete	0.13	169,770	5,122	183	Nose	HE
328	CP077897	SAMN19550041	2,764,234	1	2,764,234	33	2,594	59	Complete	0.13	243,189	5,188	254		
329	CP077894 CP077895 CP077896	SAMN19550042	2,877,025	3	2,795,787	32.9	2,659	59	Complete	0.78	158,893	5,223	251		
332	CP077893	SAMN19550043	2,745,575	1	2,745,575	32.8	2,579	59	Complete	0.08	126,671	5,338	349	Nose	HE
333	CP077889 CP077890 CP077891 CP077892	SAMN19550044	2,840,121	4	2,745,576	32.8	2,621	59	Complete	3.45	195,715	4,983	298		
334	CP077888	SAMN19550045	2,745,573	1	2,745,573	32.8	2,584	59	Complete	0.08	156,913	5,237	395		
355	CP077885 CP077886 CP077887	SAMN19550046	2,912,691	3	2,855,354	32.7	2,781	58	Complete	0.17	147,539	4,626	312	Nose	AE
356	CP077880 CP077881 CP077882 CP077883 CP077884	SAMN19550047	2,930,327	5	2,855,358	32.7	2,799	58	Complete	0.16	169,612	3,707	356		
357	CP077877 CP077878 CP077879	SAMN19550048	2,898,286	3	2,855,353	32.7	2,770	58	Complete	0.12		4,959	310		
358	CP077872 CP077873 CP077874 CP077875 CP077876	SAMN19550049	2,926,152	5	2,855,863	32.7	2,790	58	Complete	0.16	497,238	4,670	284	Skin	
359	CP077933 CP077934 CP077935	SAMN19550050	2,884,333	3	2,599,728	32.7	2,751	56	Complete	0.12	95,473	4,369	145		
360	CP077870 to CP077871	SAMN19550051	2,902,129	2	2,855,872	32.8	2,768	58	Complete	0.08	120,834	4,904	339		
365	CP077869	SAMN19550052	2,757,494	1	2,757,494	32.9	2,553	59	Complete	0.22	120,639	4,256	236	Nose	AE
366	CP077868	SAMN19550053	2,757,281	1	2,757,281	32.9	2,552	57	Complete	0.22	98,907	4,611	390		
367	CP077867	SAMN19550054	2,786,390	1	2,786,390	32.9	2,571	57	Complete	0.78	135,207	5,389	405		
370	CP077865 CP077866	SAMN19550055	2,838,117	2	2,756,732	32.9	2,609	59	Complete	4.05	138,422	5,100	465	Skin	
371	CP077863 CP077864	SAMN19550056	2,793,848	2	2,757,339	32.9	2,566	59	Complete	0.22	179,644	5,129	582		
372	CP077861 CP077862	SAMN19550057	2,798,879	2	2,757,024	32.9	2,576	59	Complete	1.91	169,836	5,166	918		
AC4	CP077860	SAMN19550058	2,766,976	1	2,766,976	32.9	2,599	59	Complete	0.08	206,880	5,165	638	Skin, nonlesional, AC^*b*^	AE
AC5	CP077859	SAMN19550059	2,766,976	1	2,766,976	32.9	2,599	59	Complete	0.08	196,190	5,092	312		
AC6	CP077858	SAMN19550060	2,766,990	1	2,766,990	32.9	2,597	59	Complete	0.08	281,434	4,691	412		
L4	CP077857	SAMN19550061	2,766,975	1	2,766,975	32.9	2,599	59	Complete	0.08	181,163	4,842	278	Skin, lesional, hand	
L5	CP077856	SAMN19550062	2,766,975	1	2,766,975	32.9	2,597	59	Complete	0.08	178,032	4,834	221		
L6	CP077855	SAMN19550063	2,766,978	1	2,766,978	32.9	2,597	59	Complete	0.08	95,473	5,045	357		
N4	CP077854	SAMN19550064	2,766,976	1	2,766,976	32.9	2,598	59	Complete	0.08	200,607	4,866	276	Nose	
N5	CP077853	SAMN19550065	2,766,976	1	2,766,976	32.9	2,599	59	Complete	0.08	152,840	5,114	273		
N6	CP077852	SAMN19550066	2,766,978	1	2,766,978	32.9	2,599	59	Complete	0.08	232,973	4,993	306		

a AE, patient with atopic eczema; HE, healthy control person.

b AC, antecubital fossa.

### Data availability.

The genome assemblies have been deposited in the DDBJ/ENA/GenBank databases under the accession numbers CP077852 to CP077932. The raw reads (SAMN19550019 to SAMN19550066) have been deposited in the Sequence Read Archive under the BioProject accession number PRJNA732579.
